# Is a voluntary healthy food policy effective? evaluating effects on foods and drinks for sale in hospitals and resulting policy changes

**DOI:** 10.1186/s12916-025-04122-x

**Published:** 2025-05-28

**Authors:** Cliona Ni Mhurchu, Magda Rosin, Stephanie Shen, Bruce Kidd, Elaine Umali, Yannan Jiang, Sarah Gerritsen, Sally Mackay, Lisa Te Morenga

**Affiliations:** 1 Department of Epidemiology and Biostatistics, School of Population Health, Faculty of Medical and Health Sciences, University of Auckland, Private Bag 92019, Auckland Mail Centre, Auckland, 1142 New Zealand; 2 Centre for Translational Health Research: Informing Policy and Practice (TRANSFORM), Faculty of Medical and Health Sciences, University of Auckland, Auckland, New Zealand; 3 National Institute for Health Innovation, School of Population Health, Faculty of Medical and Health Sciences, University of Auckland, Auckland, New Zealand; 4 Department of Statistics, Faculty of Science, University of Auckland, Auckland, New Zealand; 5 Department of Social and Community Health, School of Population Health, Faculty of Medical and Health Sciences, University of Auckland, Auckland, New Zealand; 6 Research Centre for Hauora and Health, Massey University, Wellington, New Zealand

**Keywords:** Healthy food guidelines, Nutrition, Hospital, Audit, Evaluation, Digital tool, Implementation

## Abstract

**Background:**

Healthy food and drink guidelines for public sector settings can improve the healthiness of food environments. This study aimed to assess the implementation and impact of the voluntary National Healthy Food and Drink Policy (the Policy) introduced in New Zealand in 2016 to encourage provision of healthier food and drink options for staff and visitors at healthcare facilities.

**Methods:**

A customised digital audit tool was used to collate data on foods and drinks available for sale in healthcare organisations and to systematically classify items as green (‘*healthy*’), amber (‘*less healthy*’), or red (‘*unhealthy*’) according to Policy criteria. On-site audits were undertaken between March 2021 and June 2022 at 19 District Health Boards (organisations responsible for providing public health services) and one central government agency. Forty-three sites were audited, encompassing 229 retail settings (serviced food outlets and vending machines). In total, 8485 foods/drinks were classified according to Policy criteria. The primary outcome was alignment with Policy guidance on the availability of green, amber, and red category food/drink items (≥ 55% green and 0% red items). Secondary outcomes were proportions of green, amber, and red category items, promotional practices, and price. Chi-square tests were used to compare results between categorical variables.

**Results:**

No organisation met the criteria for alignment with the Policy. Across all sites, 38.9% of food/drink items were rated red (not permitted), 39.0% were amber, and 22.1% were green. Organisations that adopted the voluntary Policy offered more healthy foods/drinks than those with their own organisational policy, but the proportion of red items remained high: 32.3% versus 47.5% (*p* < 0.0001). About one-fifth (21.3%) of all items were promoted, with red (24.6%) and amber (22.2%) items significantly more likely to be promoted than green items (14.0%) (*p* < 0.001). Green items were also significantly more costly on average (NZ$6.00) than either red (NZ$4.00) or amber (NZ$4.70) items (*p* < 0.0001).

**Conclusions:**

Comprehensive and systematic evaluation showed that a voluntary Policy was not effective in ensuring provision of healthier food/drink options in New Zealand hospitals. The adoption of a single, mandatory Policy, accompanied by dedicated support and regular evaluations, could better support Policy implementation.

**Supplementary Information:**

The online version contains supplementary material available at 10.1186/s12916-025-04122-x.

## Background

Globally [1], and in New Zealand [2–4], food environments are predominated by food and drinks that do not support healthy dietary choices [5], contributing to high rates of obesity and diet-related diseases [6]. Improving the healthiness of food environments requires interventions at multiple levels and across multiple domains [7], as well as collaboration among many parties and a substantial investment of time and resources due to the complex nature of such initiatives [8]. The NOURISHING framework recommends 10 actions to promote healthy diets across three domains: the food environment, the food system, and behaviour change [9]. Recommended food environment actions include healthy food provision in public sector settings (such as healthcare facilities, workplaces, and schools) and healthy retail and food service environments. Healthy food and drink policies and guidelines can play an important role in improving food environments [10, 11], but they are often voluntary. However, research shows that voluntary initiatives, including front-of-pack food labelling schemes aimed at supporting consumers in making healthier choices [12], are less effective than regulatory approaches [13, 14].

A National Healthy Food and Drink Policy (the Policy) was introduced in New Zealand in 2016 with the intention of supporting and encouraging the provision of healthier food and drink options for staff and visitors in hospitals and public sector organisations [15]. Adoption of the Policy allowed health sector organisations to demonstrate their commitment to supporting health and well-being of staff, visitors, and the wider community, reinforce their role in modelling healthy eating, and communicate consistent criteria for the food industry operating within the New Zealand health sector [16]. The Policy was developed by the National Healthy Food and Drinks Environments Network (the Network), an alliance of nutrition, dietetic, food service, and/or public health representatives from all 20 District Health Boards (DHBs, organisations responsible for providing or funding the provision of health services in their district) across New Zealand along with the Ministry of Health [15]. The Network was formed in 2015 with the objective of establishing one set of consistent standards to be used across all 20 DHBs [15]. Before then, DHBs had been developing and implementing various different food and drink standards [17]. The Policy was published in 2016 and was intended to be implemented over the following 2 years [15]. A second edition of the Policy was released in 2019 after a limited review [16]. However, no future reviews of the Policy were specified at the time.

The Policy provides guidance on healthy food and drink principles (based on the Eating and Activity Guidelines for New Zealand Adults [18]) and uses a colour-coded categorisation system to classify foods and drinks as green, amber, or red. The Policy covers foods and drinks sold or provided to staff and visitors on-site by internal or external food providers, or off-site on behalf of the organisation. The green items are *healthy*, everyday and high nutritional quality options and reflect a variety of foods/drinks that are mostly whole and less processed and low in saturated fat, added sugar and added salt (such as fresh and frozen fruit and vegetables, eggs, unsalted nuts and seeds, plain low-fat milk, and high fibre and low sodium bread) [16]. The amber items are *less healthy*, more processed and higher in added sugar, salt and/or saturated fat than healthy options; however, they may still provide some nutritional value and are often restricted to smaller portion sizes (e.g. dried fruits, salted nuts, full-fat dairy products, refined grains, bakery items, and non-added sugar cold drinks) [16]. The red items are *unhealthy*, highly processed foods/drinks (such as deep-fried foods, confectionery, and sugar-sweetened beverages) with very little to no nutritional value [16].

The Policy uses a combination of specific criteria to differentiate between green, amber, and red options within eight main food categories and one cold drink category. These criteria include ingredients/content (e.g. added sugar or low-fat), processing level, cooking method, nutrient levels per 100 g/mL and/or per packet, serving size limits, and front-of-pack voluntary labelling Health Star Rating (HSR) system scores. Most packaged foods need to meet a minimum threshold of ≥ 3.5 HSR stars (out of possible 5 stars) for green and amber products [16]. The Policy states that green foods and drinks ‘should make up at least 55 percent of food and drinks available for consumption’, amber items should ‘make up less than 45% of choices available’, and ‘red items are not permitted’ ([16], p. 8). Additionally, green items should be the most prominently displayed, actively promoted, and competitively priced (in contrast to amber items) [16]. However, the traffic light criteria are intended to only act as a guide to increase the overall availability of healthier options, rather than as a point-of-purchase promotional tool or a healthiness indicator for customers [16].

The Policy is however voluntary. Whilst DHBs were encouraged to adopt it, there was no regulatory requirement for them to do so, and many elected to keep or develop their own organisational food policy. An assessment of New Zealand institutional healthy food and drink policies published in 2022 found that of 22 organisations assessed (20 DHBs and two national government agencies), eight DHBs and one agency reported adopting the Policy in full [19]. Of the remaining 13 organisations, six referenced the Policy when developing their institutional policy, and three were working towards full adoption of the Policy.

By 2021, the Policy was in place for 5 years and a comprehensive, independently funded evaluation (the HealthY Policy Evaluation (HYPE)) was undertaken to assess its overall implementation and impact (see citations to other HYPE studies [19–26]). This paper reports on one component of the HYPE study, which was to assess alignment with the Policy of foods and drinks available or offered for sale to staff and visitors in New Zealand DHBs and central government organisations.

## Methods

### Study design and settings

The Policy was developed to encourage provision of healthier food and drink options in New Zealand hospitals and public sector organisations. The objective of this cross-sectional, observational study was to audit the foods and drinks available for sale in all New Zealand DHBs (20 in total) and one central government agency (the Ministry of Health). Between March 2021 and June 2022, comprehensive on-site audits covered all food retail settings at participating organisation sites, available catering offered on organisational premises, and other areas that provided access to foods or drinks for staff or visitors. Food retail outlets included café kiosks and coffee carts, cafés and cafeterias, vending machines, sushi outlets, on-site fundraisers, pop-up markets, and other retail outlets such as pharmacies, gift shops, and snack box vendors. The time of day that the audits took place was generally consistent across institutions to capture the largest amount of foods and drinks on display at a single time (usually between mid-morning and the start of lunch service).

### Data collection

Data recorded for each audited food and drink item included (where available) product name or description, brand or manufacturer name, barcode details, product and serving sizes (weight in grams (g) or volume in millilitres (mL) as given on the package or by weighing), nutrient information provided on product packaging (energy, protein, saturated fat, total sugars, sodium, and fibre content per 100 g or 100 mL, and per serve), displayed HSR value (between 0.5 and 5 stars), ingredients list, recipes for cooked mixed dishes, item availability (for packaged items, defined as the number of outward front-facing items when placed on the edge of the shelf or the number of slots an item occupies in a vending machine; for unpackaged items, defined as either available for order from a menu or pre-cooked/pre-prepared as a batch and offered instantly as individual portions), any promotional practice (price reduction, prominent display location such as eye level position, check-out display or placement near cash register), promotional sticker, feature in advertising materials, or part of a package/combo deal), unit price in NZ$, and product photos. Where nutrient information was not readily available, it was sourced from suppliers, manufacturer or supermarket websites, the Nutritrack database of packaged products [27] (managed by the University of Auckland), calculated from recipes, or the research team matched food or drink items to appropriate nutrient lines in the New Zealand food composition tables [28]. Inpatient meals, foods and drinks brought in by staff or visitors for their own consumption, and hot drinks, were excluded since these are not within the Policy scope.

**Fig. 1:**
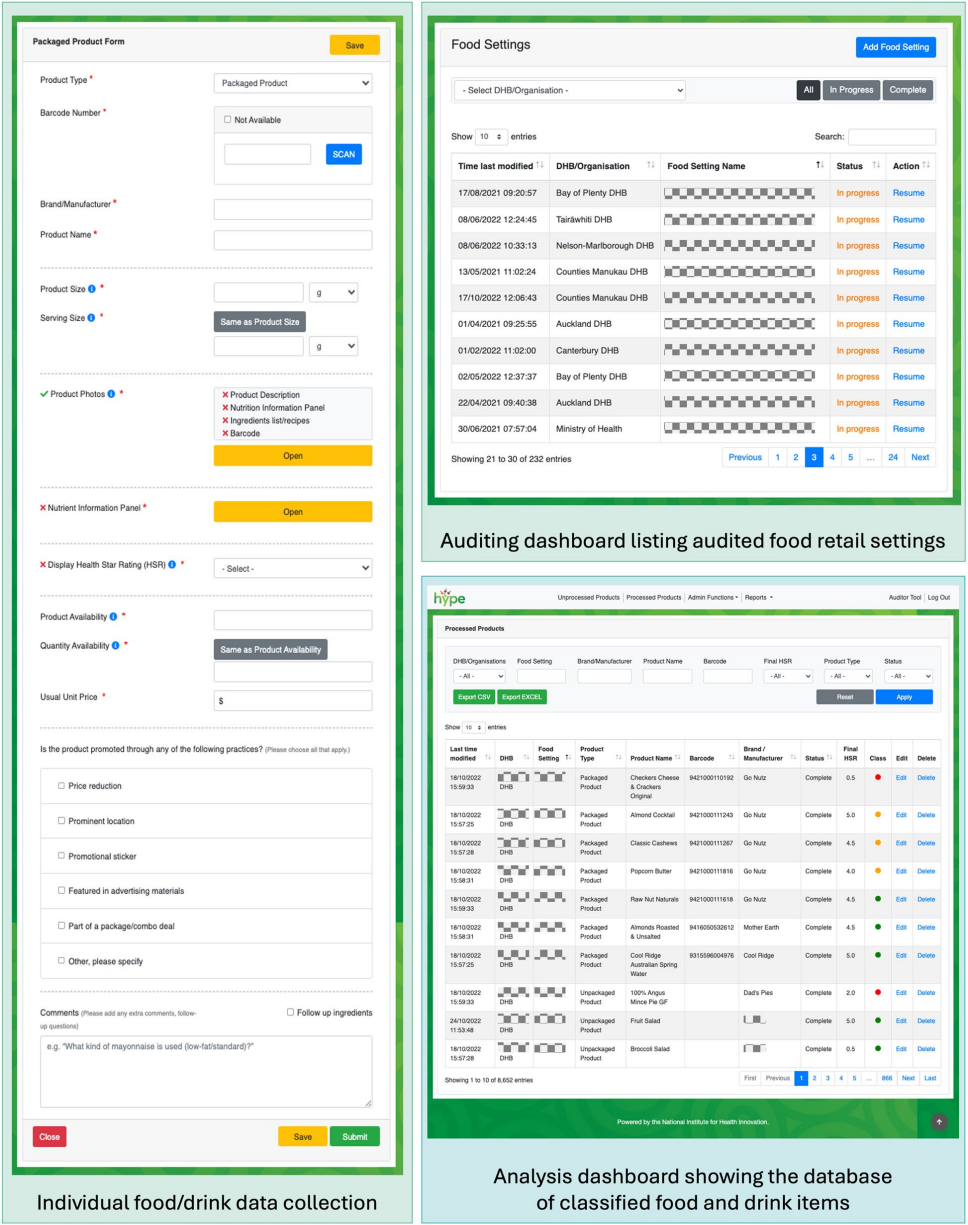
Example screenshots from the audit tool interface

A customised electronic data collection tool (HYPE audit tool) was developed on Drupal (an open-source web content management system) to record and collate information on available foods and drinks [20]. The tool was pilot-tested by the study team. The platform ensured the tool could be used on smartphones and tablets equipped with mobile data during on-site audits, as well as on laptops and desktop computers during data analysis and quality checks stages. The audit tool had the capacity to take multiple product photos, a barcode scanning function, the ability to recognise if a specific, identical food/drink item had been previously collected in another setting (minimising duplicate data collection), an incomplete data alert system (prompting researchers to follow up on information that could not be collected immediately, e.g. a recipe for a menu item), and a feature enabling quality checks on a random 10% sample of all audited food and drink products to ensure data collection was consistent and accurate. Additionally, two algorithms were incorporated into the audit tool to automatically and systematically analyse food/drink items: one to classify foods/drinks as either green, amber, or red according to the traffic light system in the Policy, and an HSR algorithm based on the publicly available HSR calculator [29] to estimate product HSR from the nutrient information data where HSR was required for the green/amber/red classification. Example screenshots from the HYPE audit tool’s user-facing screens are presented in Fig. 1.

**Fig. 2:**
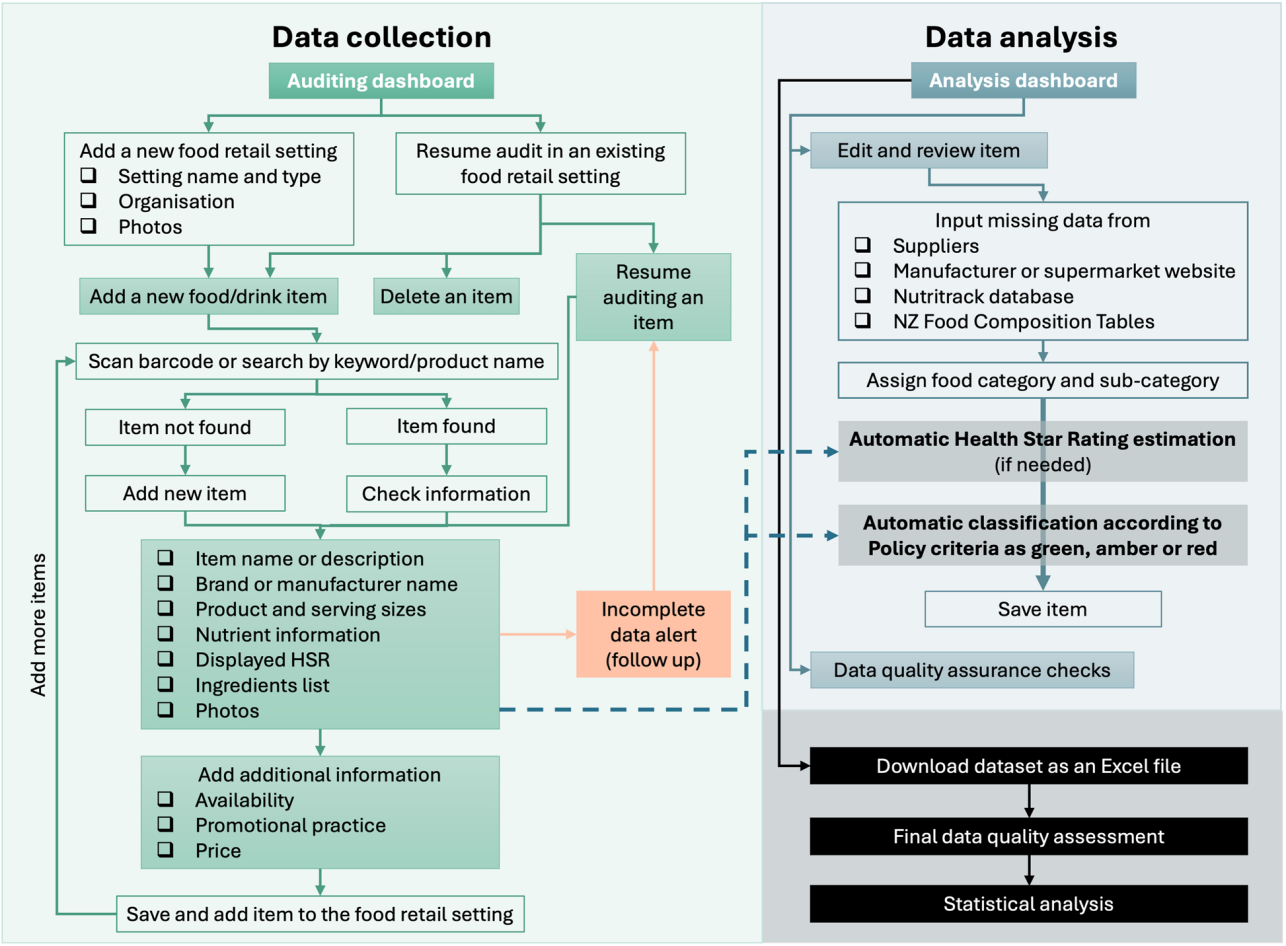
Food and drink data collection and classification framework

Each food retail setting was evaluated as a separate outlet, even if operated by the same food provider. Consequently, each food and drink item in every setting was counted, regardless of whether it was also available in another location. Data were collected by three research assistants who were trained in how to follow a data collection protocol. To reduce the data collection burden, information previously collected on the same item in another setting (product name, brand or manufacturer name, barcode, product and serving sizes, nutrient information, displayed HSR, ingredients list, and product photos) was checked for accuracy and automatically inputted from the tool’s backend into the relevant product fields. During the traffic light classification stage, each product and its ingredients were reviewed to assign it to a food/drink category and sub-category informed by the Policy criteria, which allowed for systematic classification of the items in the database based on the product/serve sizes, nutrient values, and/or HSR rating (see Additional file 1: Table S1). Any changes made to the data following the quality checks in the audit tool were reviewed and agreed upon by two study authors (SS, BK). The full and final dataset, including classification by organisation and food retail setting, was downloaded from the audit tool as an Excel spreadsheet. A final data quality assessment check was carried out by two authors (MR, SM). The food and drink data collection and classification framework is outlined in Fig. 2.

### Outcomes

The primary outcome of interest was implementation of the Policy by organisations, specifically, the alignment of food and drink availability on-site with Policy guidelines, where alignment was defined as ≥ 55% green category items and 0% red category items.

Secondary outcomes were proportions of green, amber, and red category items (compared between regions, organisations, and Policy adoption status); food marketing and promotion practices on-site; and the mean price of green, amber, and red foods and drinks available.

### Statistical analysis

Audit data were imported from a secure study database to SAS version 9.4 (SAS Institute Inc., Cary, NC, USA) for analysis. Analyses were based on unique products, i.e. products were counted only once, irrespective of how many instances of a product were present in an individual retail setting. Primary and secondary outcomes were summarised descriptively as frequencies and percentages. The proportions of green, amber, and red category items were compared between groups (e.g. organisation, policy) using chi-square tests at 5% significance level. Missing data were noted and excluded from the analysis. Specifically, any item with incomplete information needed to classify it as red, amber, or green was removed from the dataset using listwise deletion.

## Results

### Characteristics of participating organisations

Nineteen (from a total of 20) DHBs and the Ministry of Health provided locality approval for the on-site audits, leading to a final sample of 20 organisations representing all areas and the four major regions (Northern, Te Manawa Taki, Central, and Te Waipounamu) of New Zealand. The regions were denoted with letters A to D and the participating organisations with numbers 1 to 20. Regions and organisations were anonymised to protect the privacy and confidentiality of the food outlet operators. The characteristics of participating organisations are outlined in Table 1. In 2022, the estimated population serviced by the 19 DHBs was 4,671,990 or 91.2% of the total New Zealand population [30]. Individual DHB populations ranged from 32,700 to 633,500. DHBs employed approximately 80,000 staff in 2021, 22.3% of whom were Asian, 8.5% Māori, 4.6% Pacific, and 64.6% New Zealand/Other [31]. The Ministry of Health employed over 800 staff based in 6 locations across New Zealand in 2022 [32].

**Table 1:** Characteristics of participating organisations

Region	Organisation	Population served (2022)	Number of operating sites	Number of food retail settings	Food retail setting types						
					Café kiosks and coffee carts	Cafés and cafeterias	Vending machines	Sushi outlets	On-site fundraisers	Pop-up markets	Other retail outlets^a^
All organisations and regions combined		4,671,990	43	229	11	64	138	3	2	1	10
A	01	201,500	2	6	1	3	2	–	–	–	–
	02	633,500	2	18	2	5	11	–	–	–	–
	03	481,600	4	49	3	8	37	1	–	–	–
	04	605,100	2	28	3	6	16	2	–	1	–
B	05	118,200	2	8	–	3	3	–	–	–	2
	06	274,700	2	9	–	3	6	–	–	–	–
	07	52,100	1	1	–	1	–	–	–	–	–
	08	127,500	2	9	1	2	6	–	–	–	–
C	09	182,600	2	10	–	2	6	–	–	–	2
	10	69,500	1	3	–	2	1	–	–	–	–
	11	190,300	2	10	–	3	6	–	–	–	1
	12	160,200	1	8	–	2	6	–	–	–	–
	13	322,300	3	19	1	5	10	–	1	–	2
	14	51,000	1	3	–	1	2	–	–	–	–
D	15	165,000	2	2	–	2	–	–	–	–	–
	16	32,700	2	2	–	1	–	–	–	–	1
	17	591,500	6	30	–	8	20	–	1	–	1
	18	62,300	1	3	–	1	1	–	–	–	1
	19	350,500	4	10	–	5	5	–	–	–	–
National	20	N/A	1	1	–	1	–	–	–	–	–

^a^Category includes pharmacies, gift shops, and snack box vendors

In total, 43 sites were audited, comprising 229 individual retail settings (café kiosks and coffee carts, cafés and cafeterias, vending machines, sushi outlets, on-site fundraisers (where they occurred during audits), pop-up markets, and other retail outlets) (Table 1). The majority of retail settings were vending machines (*n* = 138, 60.3%) and cafés or cafeterias (*n* = 64, 27.9%). Information was collected for 8651 foods and drinks available for sale across all sites and settings. One hundred and sixty-six products (1.9%) could not be classified as red, amber, or green according to the Policy criteria due to missing nutrition information, leading to a final audit sample of 8485 products. Of these, 2486 products (29.3%) were available from vending machines, 4782 products (56.4%) were from cafés or cafeterias, and 1217 products (14.3%) were from other food outlets.

### Primary outcome

Alignment of on-site food availability with the Policy was defined as ≥ 55% green category items and 0% red category items. No organisation met the criteria for alignment (Table 2).

**Table 2:** Classification of food and drink items available by region and organisation

Region	Organisation	Total number of products with classification	Classification					
			Red		Amber		Green	
		*n*	*n*	%	*n*	%	*n*	%
All organisations and regions combined		8485	3303	38.9	3304	39.0^a^	1878	22.1
A	01	280	73	26.1	97	34.6	110	39.3
	02	778	188	24.2	408	52.4	182	23.4
	03	1797	514	28.6	948	52.8	335	18.6
	04	1151	410	35.6	524	45.5	217	18.9
	Total	4006	1185	29.6	1977	49.4	844	21.1
B	05	165	88	53.3	36	21.8	41	24.8
	06	372	136	36.6	146	39.2	90	24.2
	07	56	16	28.6	24	42.9	16	28.6
	08	349	224	64.2	72	20.6	53	15.2
	Total	942	464	49.3	278	29.5	200	21.2
C	09	362	188	51.9	112	30.9	62	17.1
	10	162	67	41.4	59	36.4	36	22.2
	11	292	139	47.6	95	32.5	58	19.9
	12	188	60	31.9	69	36.7	59	31.4
	13	702	433	61.7	165	23.5	104	14.8
	14	79	24	30.4	32	40.5	23	29.1
	Total	1785	911	51.0	532	29.8	342	19.2
D	15	139	46	33.1	58	41.7	35	25.2
	16	86	30	34.9	26	30.2	30	34.9
	17	916	451	49.2	275	30.0	190	20.7
	18	98	49	50.0	29	29.6	20	20.4
	19	438	136	31.1	108	24.7	194	44.3
	Total	1677	712	42.5	496	29.6	469	28.0
National	20	75	31	41.3	21	28.0	23	30.7

^a^Amber items percentage was the largest rounded percentage and was adjusted by 0.1% to address rounding discrepancy

### Secondary outcomes

#### Proportions of green, amber, and red category items

Across all sites, an average of 38.9% of food/drink items available for sale were classified as red (not permitted), 39.0% were amber (amber was the largest rounded percentage and was adjusted by 0.1% to address rounding discrepancy), and just 22.1% were green. The prevalence of red items available at individual organisations ranged from 24.2% (Organisation 02) to 64.2% (Organisation 08) (Table 2). The highest proportion of green items available at any organisation was 44.3% (Organisation 19). There were notable differences by geographic area, with region A having significantly fewer red food/drink items available (29.6%) compared with regions B (49.3%), C (51.0%), and D (42.5%) (*p* < 0.0001).

Eight of the 20 organisations had adopted the national Policy whilst 12 were using their own organisational healthy food policy or guidelines [19]. Of these 12, some stated that the Policy was a basis for their organisational policy, whilst others said that they intended to adopt the Policy in future. Organisations that had adopted the Policy had better alignment with the Policy criteria than those with their own organisational policy (*p* < 0.0001), but the proportion of red items available at such organisations was still substantial (32.3% in Policy organisations versus 47.5% in organisations with their own policy), and the proportion of green items was not different (22.2% versus 22.0%) (Table 3).

**Table 3:** Classification of food and drink items available according to adoption of national Policy

Adopted national Policy	Organisation	Total number of products with classification	Classification					
			Red		Amber		Green	
		*n*	*n*	%	*n*	%	*n*	%
No	01	280	73	26.1	97	34.6	110	39.3
	05	165	88	53.3	36	21.8	41	24.8
	06	372	136	36.6	146	39.2	90	24.2
	07	56	16	28.6	24	42.9	16	28.6
	08	349	224	64.2	72	20.6	53	15.2
	10	162	67	41.4	59	36.4	36	22.2
	11	292	139	47.6	95	32.5	58	19.9
	12	188	60	31.9	69	36.7	59	31.4
	13	702	433	61.7	165	23.5	104	14.8
	14	79	24	30.4	32	40.5	23	29.1
	15	139	46	33.1	58	41.7	35	25.2
	17	916	451	49.2	275	30.0	190	20.7
	Total	3700	1757	47.5	1128	30.5	815	22.0
Yes	02	778	188	24.2	408	52.4	182	23.4
	03	1797	514	28.6	948	52.8	335	18.6
	04	1151	410	35.6	524	45.5	217	18.9
	09	362	188	51.9	112	30.9	62	17.1
	16	86	30	34.9	26	30.2	30	34.9
	18	98	49	50.0	29	29.6	20	20.4
	19	438	136	31.1	108	24.7	194	44.3
	20	75	31	41.3	21	28.0	23	30.7
	Total	4785	1546	32.3	2176	45.5	1063	22.2

**Fig. 3:**
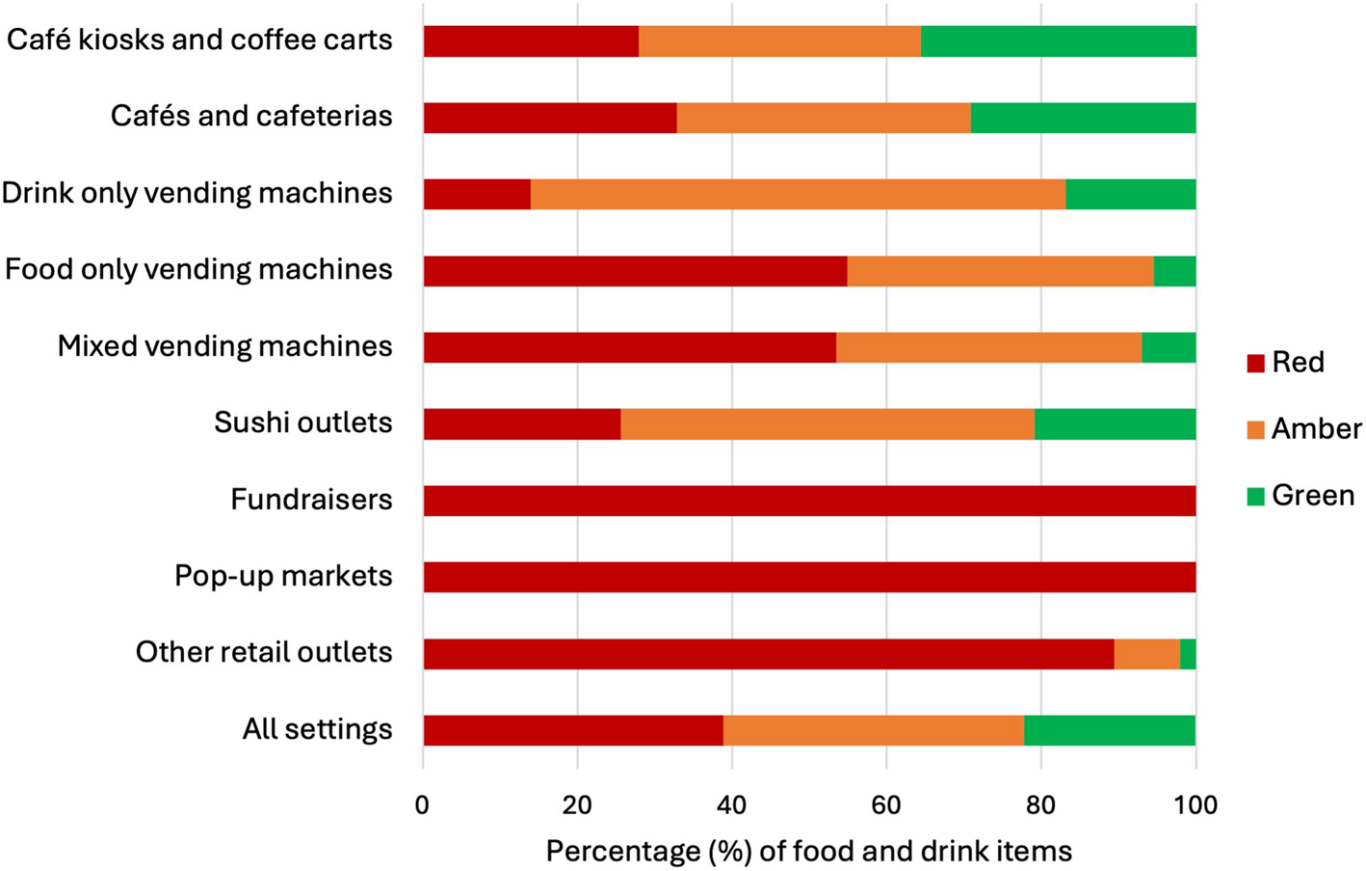
Classification of food and drink items available by retail setting

The healthiness of foods and drinks varied substantially by retail setting (Fig. 3). Drink-only vending machines contained the fewest red items (14.0%) compared with other retail settings, where the prevalence of red items ranged from 25.6% (sushi outlets) to 100% (on-site fundraisers) (*p* < 0.0001). Over half the products available in food-only vending machines and mixed food and drink vending machines were red items (54.9% and 53.5%, respectively), and almost all items sold in fundraisers, ‘other’ retail outlets (pharmacies, gift shops, and snack box vendors), and pop-up markets were red (100%, 89.4%, and 100%, respectively).

**Fig. 4:**
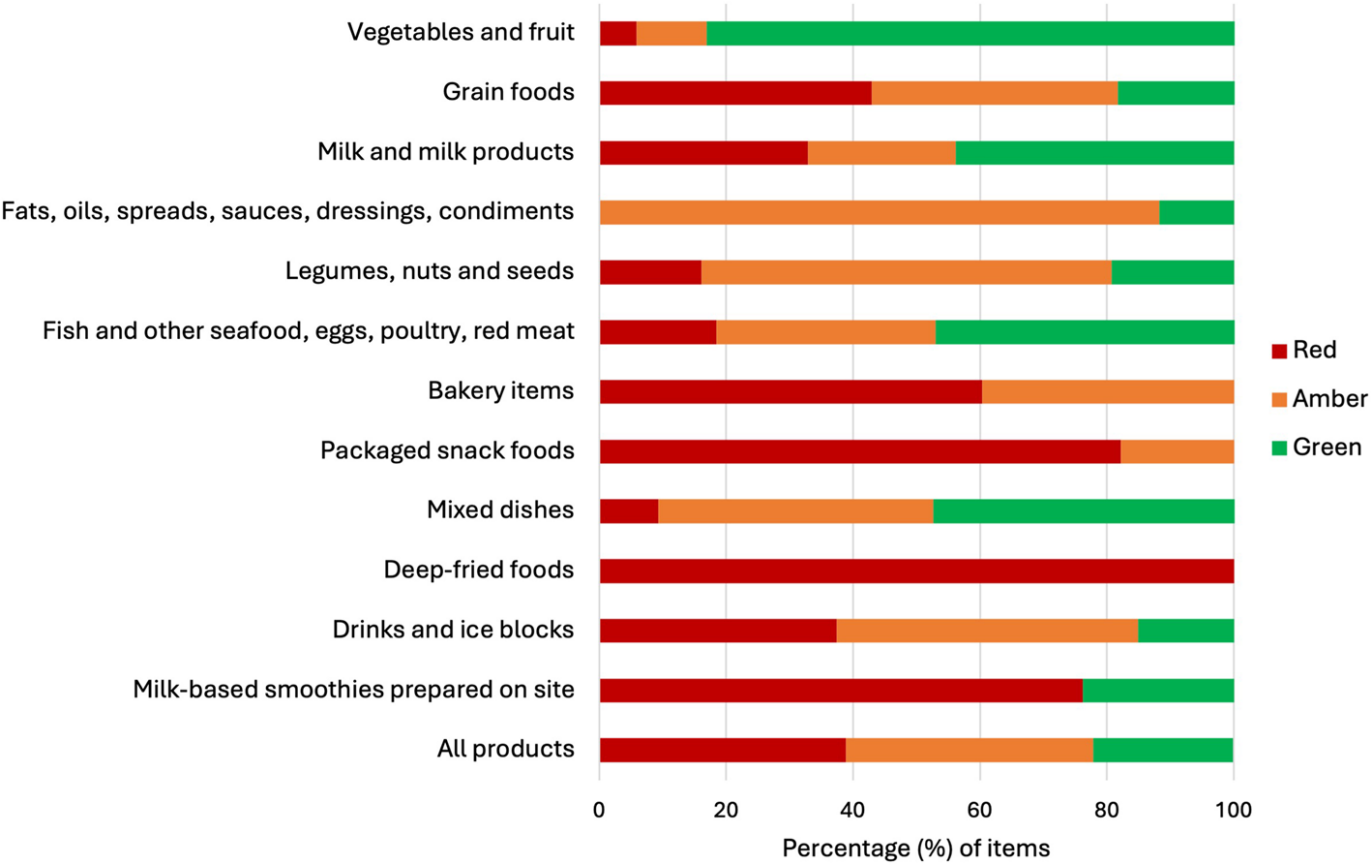
Classification of food and drink items available by Policy category

Product healthiness also varied by the Policy food category (Fig. 4). There were six instances of deep-fried foods identified during audits, all of which were red items (not permitted). In addition, 82.2% of packaged snack foods were classified as red, as were 60.3% of bakery items. These latter two categories contained substantially more products than other food/drink categories and together accounted for 3090 products or over one-third (36.4%) of all products audited. The Policy criteria for bakery items and packaged snack foods mean it is not possible for any items in these categories to be classified as green (since no green criteria have been specified); nevertheless, the predominance of red versus amber items is a concern.

#### Food marketing and promotion practices

The Policy states that ‘“Green item” foods and drinks should be the most prominently displayed items by retailers and should be readily available in sufficient quantities, competitively priced, and promoted to encourage selection of these options’ [16], p. 5. Most food/drink items audited were not promoted, but about one-fifth (21.3%) were, with red (24.6%) and amber (22.2%) items significantly more likely to be on promotion than green items (14%) (*p* < 0.0001). Food items most likely to be classified as on promotion (mainly due to placement in a prominent location) were ‘fats and oils, spreads, sauces, dressings, condiments’ (49.8% such products were placed near cash registers) and ‘bakery items’ (32.6% bakery items). The items least likely to be classified as on promotion were ‘milk and milk products’ (2.4%) and ‘mixed dishes’ (9.2%), such as rice- or pasta-based meals, sandwiches, soups, salads, and sushi. Green items were significantly more costly on average per item (NZ$6.00) than either red (NZ$4.00) or amber (NZ$4.70) foods/drinks (*p* < 0.0001). For brevity purposes, detailed pricing results are not reported here. However, additional details can be made available upon reasonable request, in line with the study’s ethical approvals and data sharing restrictions.

## Discussion

We assessed the implementation and impact of the National Healthy Food and Drink Policy, a voluntary policy introduced in New Zealand in 2016 to support and encourage the provision of healthier food and drink options in hospitals and health sector organisations. On-site audits undertaken at 20 organisations in 2021/22 showed that none met the Policy criteria in terms of proportions of green, amber, and red items available to staff and/or visitors. Almost two in five (38.9%) of food/drink choices available for staff and/or visitors were red items, which the Policy states are not permitted. In contrast, less than one in four (22.1%) were green items, which the Policy specifies should make up at least 55% of food and drinks available for consumption. Whilst those organisations that had adopted the Policy demonstrated better alignment with the Policy compared to those that had not adopted it, no organisation met the guidance on not providing red items. Thus, almost 5 years after its introduction, the Policy did not meet its objective of ensuring that healthier food and drink options make up the majority of choices available in hospitals and health sector organisations.

Possible reasons for our findings are as follows: first, the Policy was voluntary and any consequences for non-adherence were at the discretion of individual organisations. The World Health Organization states that ‘effective (policy) criteria are mandatory, specific and enforceable, and applicable to all government food purchases and all food served or sold in public settings’ [33], p. v. In this case, it was up to individual organisations to decide whether to adopt the Policy and there were no enforcement mechanisms. Second, no additional resources were allocated to support the development, implementation, and evaluation of the Policy, nor to engage with and secure buy-in from stakeholders [21]. These tasks were largely undertaken within the scope of existing hospital dietitian or health promotion advisor roles, often as additional work commitments [22].

Third, even where organisations adopted the Policy at an executive level, retailers and food service providers may not have complied with the Policy due to concerns that adherence could impact adversely on food sales [23, 34, 35]. Interviews with food providers in New Zealand hospitals, conducted as part of the HYPE study, highlighted potential loss of profits as a major barrier to implementation [22]. This barrier was associated with food outlets near the hospitals that were not covered by the Policy selling unhealthy foods, inconsistent customer demand for healthier options, and anticipated increases in food waste from unsold Policy-compliant healthier products [22]. Additionally, whilst staff and visitors to New Zealand hospitals generally supported having the Policy, positive feedback on the increase in healthier options was outweighed by concerns about reduced value for money (with green items identified as the most costly per item in this study) and more limited choice [24]. Overall, the HYPE study findings suggest the need for strategies to mitigate financial risks for food providers and ensure market availability of affordable healthy options.

Fourth, retailers and food service providers may have struggled to interpret and apply complex Policy criteria, particularly since classification of some packaged foods as amber or green relies on meeting a minimum Health Star Rating (HSR) threshold (3.5 stars), often along with additional criteria such as serving size thresholds [16]. However, the HSR front-of-pack labelling system is voluntary and HSR labels are displayed on fewer than one-third of packaged foods in New Zealand [36]. Of the 8485 products audited and classified in this evaluation, HSR was displayed on just 17.9%. Although there is a calculator available to assist with estimation of HSRs for foods, the calculation itself is complex and requires knowledge of food components that are not always listed on Nutrition Information Panels (fibre and fruit, vegetable, nut, and legume content). Such issues can be overcome if resources such as dedicated technical expertise or centralised databases of classified products are available to support policy implementation [23, 35]; however, no such tools were offered with this Policy [21].

Additionally, independent auditing of foods and drinks available in New Zealand hospitals was rare and inconsistent, even though regular monitoring can indicate progress as well as identify areas where more support is needed [37]. Sporadic evaluations were likely due to non-existent or limited capacity in each DHB to undertake this additional work [22], amplified by a lack of standardised and systematic audit tools [21]. Commonly used methods for monitoring public sector food environments include paper-based audit tools, photographing products available in retail outlets, and manual classification of items against policy criteria [37–39]. The advantages of electronic audit tools include reduced burden of data collection and analysis, automated and systematic item classification, and storage and management of all data in a single platform [40–42]. The HYPE audit tool could be further adapted to support public health nutrition professionals in consistent and regular monitoring of the Policy [22], as well as to monitor healthy food policies in other public settings such as schools and early childhood education centres.

Finally, retailers and food service providers may have experienced challenges in sourcing healthier food/drink options due to a general lack of market demand for such products [43–45]. The fact that drink-only vending machines had fewer red items than other retail settings likely reflects a longstanding focus on healthier drink options at many DHBs (e.g. with three DHB policies specifying that only water and plain milk were allowed [19]) and more green/amber drinks options available for drinks than in other Policy categories. The difficulties in sourcing healthy and low-cost snacks suitable for vending machines could lead to low compliance or removal of food vending machines altogether [46], even though health professionals, especially those working long hours or late shifts, and visitors to hospitals, often rely on food in vending machines to supplement their diets [47]. Whilst vending machines have been often described as sources of unhealthy foods and drinks [48, 49], research shows that mandatory organisational policies [50] and interventions targeted at improving vending machine offerings [51] can increase availability and purchasing of healthier options [47]. More widespread adoption of healthy food and drink policies by public sector organisations could create greater demand for healthier products and hence motivate manufacturers to invest in the development of healthier foods and drinks [52].

Although public sector healthy food and drink policies are common, there are relatively few comprehensive evaluations of their impact on food and drink availability. In Western Australia, a state-wide audit of compliance with Healthy Options Western Australia (WA), a mandatory system-wide healthy food policy for WA health services and facilities, was undertaken in 2018/19 [39]. Under this policy, at least 50% of all food and drink on offer and on display must be classified as green, no more than 20% may be classified as red, with the remainder classified as amber [53]. Twenty-five facilities were audited comprising 217 food providers (vending machines, cafés, and ward trolleys), and over 7000 food/drink items were classified. Just over half (51%) of the 217 audited WA food providers were compliant with the policy [39]. This compares with seven of the 229 (3%) food retail settings audited in New Zealand. The higher rate of compliance in WA healthcare institutions compared with our audit findings may be because the WA policy allows up to 20% of items to be red. Detailed audit results showed that many food providers did not fully remove red items but rather kept them under the 20% threshold [39], so a strict 0% red items criterion could lower compliance rates significantly. Interviews conducted as part of the HYPE study highlighted that the Policy was perceived by food providers as too restrictive [22]. At the same time, a DHB Network member explained that whilst there was a strict compliance goal of 0% red cold drinks in their DHB, the 0% red food items criterion was not the primary goal, and instead, the implementation efforts focussed on increasing green food options [22]. However, the higher rate of compliance in WA jurisdictions could also have been because the WA Policy was mandatory, the Department of Health provided support for implementation with a suite of resources, there was leadership from the Minister of Health who requested that all facilities become compliant with the WA policy by 2018, and a promised evaluation against policy requirements [39], all of which were implementation elements missing in New Zealand.

### Implications for policy and practice

The findings of this research suggest that policies should be evaluated regularly to assess level of implementation and identify areas for improvement, and a proportion of policy funding should be set aside for this purpose. Clauses should be inserted into organisational food service provider and food retailer contracts to ensure they must abide by the Policy, an initiative previously shown to improve the nutritional quality of vending machine products [54], and regulation should be considered where voluntary policies are shown to be insufficient and ineffective. Additionally, the results from this research interpreted in the context of the wider HYPE study [19–26] suggest that policy development should be accompanied by sufficient resources to facilitate engagement with key stakeholders prior to policy adoption and to develop practical tools to support policy implementation by end users.

Our findings show that priority retail settings for improvement in New Zealand healthcare organisations are food vending machines, on-site fundraisers, and non-typical food retailers (pharmacies, gift shops, and snack box vendors), all of which offer predominantly unhealthy food and drink items. In particular, vending machine offerings should be reviewed as they are the most predominant retail food setting in New Zealand hospitals. Packaged snack foods and baked goods are priority food groups in terms of removing red items from sale and increasing the availability of healthier offerings that align with the Policy. It is important to consider why food providers may be hesitant to switch from unhealthy to healthy options. Factors such as the lower production costs and longer shelf life of unhealthy ultra-processed foods make them more profitable to sell [55], along with other reasons outlined in the discussion. However, the observed variability in healthy food and drink availability by geographic area and organisation shows that some organisations and regions are further ahead in meeting the Policy guidance (possibly those that already had relevant policies or guidelines in place prior to adoption of the Policy [56]) and could offer support to other organisations struggling to meet Policy guidance.

### Resulting changes to the national healthy food and drink policy

In 2022, a major reform of the New Zealand health system resulted in the disestablishment of the 20 individual DHBs and the establishment of Te Whatu Ora—Health New Zealand, a single national health service provider [57]. The results of this analysis and other HYPE research [19–26], along with the resulting policy and practice recommendations, have been conveyed to the DHB Network and representatives from Te Whatu Ora. Encouragingly, in 2023, Te Whatu Ora communicated its intention to adopt a single, mandatory national Policy and in-principle support for resources and tools to support adoption, implementation, and monitoring, to catalyse the Policy’s implementation and intended impact.

As part of this new direction, a working group consisting of representatives from the DHB Network, and including one of the authors (MR), was formed in June 2023 and tasked with completing a third update of the Policy. The working group conducted a comprehensive assessment and analysis of availability, healthiness, and cost of the foods/drinks on the New Zealand market that corresponded to the green, amber, and red categories, and assessed food providers near hospitals (not governed by hospital policies) and the food and drinks they sell. Additionally, discussions with retailers and food service providers led to elucidation on which Policy criteria were most feasible to implement, which criteria were challenging and why, and what environmental sustainability initiatives could be improved or introduced into an updated Policy. To ensure the revised Policy and the associated implementation plan facilitate equitable adoption and implementation, the working group has also been consulting with Māori stakeholders and equity-oriented groups. Final policy revisions are underway. The main changes include updated traffic light criteria, modification of food groups and subgroups to reflect the most commonly available items in the hospitals (such as a variety of cold and hot mixed meals, bakery items, and packaged snack foods), reduced reliance on voluntary HSR scores for determining the traffic light classification of products, and the introduction of specific measures for health, equity, affordability, and sustainability. For example, one proposed update to the traffic light criteria is the inclusion of green criteria for bakery products that align with the overall definition of the green category in the Policy. Possible examples of green bakery items include portion-restricted savoury and sweet scones and muffins that contain at least two of the following: wholegrains, fruits, vegetables, nuts, seeds, or legumes, and that are otherwise unfilled and without additional toppings. The resulting policy changes and developments signal a significant step towards improving food environments in New Zealand healthcare facilities.

### Strengths and limitations

This was a comprehensive evaluation of the implementation of the voluntary National Healthy Food and Drink Policy. Audits were undertaken at 19 of the 20 New Zealand DHBs irrespective of whether organisations had adopted the Policy or not, hence enabling comparison of the healthiness of product offerings by Policy adoption. The audits encompassed all on-site food retail and vending machines within organisations, and information was collected for all products available for sale or consumption by staff or visitors. The research was funded and conducted by independent third parties, which facilitated impartial evaluation design, data collection, analysis, and interpretation. Strict protocols governed data collection methods and quality assurance, and the web-based data collection tool used embedded algorithms to calculate HSR and classify foods according to Policy criteria, thus minimising possible errors.

Some limitations should however be considered. First, no comparable pre-Policy data were collected so it was not possible to assess if any changes had previously been made to food and drink offerings in response to Policy adoption. Second, data collection took place in 2021 and 2022 when regions in New Zealand were experiencing COVID-19 outbreaks and lockdowns, meaning it is possible that fewer food and drink items were available on-site due to reductions in visitor numbers and the requirement for non-essential staff to work from home. Third, the audits captured product availability rather than product sales and it is probable that the relative proportions of red, amber, and green items would differ if sales were considered. Fourth, the accuracy of the Policy’s traffic light food classification system was not evaluated for alignment with healthfulness described in other classification systems or in the Eating and Activity Guidelines for New Zealand Adults. Further research could explore if the Policy’s food and drink classification is consistent with other relevant systems, particularly in the context of food provision policies. Finally, recent New Zealand health system reforms make pathways to translation less clear, but the new national health system may provide opportunities to consolidate guidance and support across organisations.

## Conclusions

A voluntary National Healthy Food and Drink Policy was not effective in ensuring the provision of healthier food and drink options for staff and visitors in New Zealand hospitals and health sector organisations. Of particular concern was the notable variability in the availability of healthy foods, with significant differences in the healthiness of offerings by organisation, geographic region, and policy type (national versus individual organisational policy). A single mandatory national policy should be implemented, along with resources and tools to support food service providers and retailers, to maximise organisational adherence and reduce regional inequities in healthy food availability. Improving the healthiness of food offerings in all New Zealand healthcare settings could improve the diets and health of many thousands of staff and visitors who eat at hospitals every day.

## Supplementary Information

Additional file 1: Table S1 contains food and drink categories and sub-categories based on the National Healthy Food and Drink Policy, including criteria used to categorise items as green, amber, or red.

## Data Availability

The participants of this study did not give written consent for their data to be shared publicly due to the commercially sensitive nature of the data. However, the corresponding author may grant access to specific, anonymised datasets upon reasonable request.

## Abbreviations

DHB: District Health Board
HSR: Health Star Rating
HYPE: HealthY Policy Evaluation
