# Supplementary material for: Is a voluntary healthy food policy effective? evaluating effects on foods and drinks for sale in hospitals and resulting policy changes
Source: BMC Med. 2025 May 28;23:299. doi: 10.1186/s12916-025-04122-x (PMC12121083; doi:10.1186/s12916-025-04122-x)
Supplement: Supplementary file 1 — Additional file 1: Table S1 contains food and drink categories and sub-categories based on the National Healthy Food and Drink Policy, including criteria used to categorise items as green, amber, or red. [file 12916_2025_4122_MOESM1_ESM.docx]

**Table S1.** Food and Drink categories and sub-categories based on the National Healthy Food and Drink Policy and criteria used to categorise items as green, amber or red

| **Category** | **Sub-category** | **Criteria to categorise food/drink items as green, amber or red** | | | | | | |
| --- | --- | --- | --- | --- | --- | --- | --- | --- |
|  |  | Ingredients | Serving size | Energy content | Sugar content | Sodium content | Fibre content | HSR rating^1^ |
| Vegetables and fruit | Vegetables (fresh, frozen, canned, dried) |  |  |  |  |  |  |  |
|  | Processed vegetables |  |  |  |  |  |  | ☑ |
|  | Fruit (fresh, frozen, canned) |  |  |  |  |  |  |  |
|  | Fruit (dried) |  | ☑ |  |  |  |  |  |
|  | Processed fruits |  |  |  |  |  |  | ☑ |
| Grain foods | Bread | ☑ |  |  |  | ☑ | ☑ |  |
|  | Crackers | ☑ |  |  |  |  |  | ☑ |
|  | Breakfast cereals | ☑ |  |  | ☑ |  |  | ☑ |
|  | Cereals foods (e.g., rice, pasta) | ☑ |  |  |  |  |  | ☑ |
| Milk and milk products | Milk and milk alternatives | ☑ |  |  |  |  |  | ☑ |
|  | Yoghurt and dairy food | ☑ | ☑ |  |  |  |  | ☑ |
|  | Custard | ☑ | ☑ |  |  |  |  | ☑ |
|  | Cheese | ☑ | ☑ |  |  |  |  | ☑ |
|  | Cream, sour cream, cream cheese | ☑ |  |  |  |  |  |  |
|  | Frozen desserts |  | ☑ |  |  |  |  | ☑ |
| Legumes, nuts and seeds | Legumes |  |  |  |  |  |  |  |
|  | Nuts and seeds (plain) |  |  |  |  |  |  |  |
|  | Nuts and seeds (with added salt, sugar, confectionery) | ☑ | ☑ |  |  |  |  |  |
| Fish, other seafood, eggs, poultry, red meat | Fish, seafood, poultry, meat | ☑  (fat and skin) |  |  |  |  |  |  |
|  | Eggs |  |  |  |  |  |  |  |
|  | Canned fish, chicken and meat |  |  |  |  |  |  | ☑ |
|  | Processed fish, poultry and meat | ☑ |  |  |  |  |  |  |
|  | Dried meat products |  |  | ☑ |  |  |  | ☑ |
|  |  |  |  |  |  |  |  |  |
| Fats and oils, spreads, sauces, dressings, condiments | Fats and oils | ☑ |  |  |  |  |  |  |
|  | Spreads | ☑ | ☑ |  |  |  |  |  |
|  | Nut butters | ☑ |  |  |  |  |  |  |
|  | Coconut milk and cream | ☑ |  |  |  |  |  |  |
|  | Sauces and dressings | ☑ |  |  |  |  |  |  |
|  | Savoury condiments | ☑ |  |  |  |  |  |  |
|  | Herbs and spices |  |  |  |  |  |  |  |
|  | Sweet condiments (e.g. jam) | ☑ | ☑ |  |  |  |  |  |
|  | Deep-fried foods |  |  |  |  |  |  |  |
| Packaged snack foods | Snacks (if not single ingredient foods listed in other categories) |  |  | ☑ |  |  |  | ☑ |
|  | Confectionery |  |  |  |  |  |  |  |
| Bakery items | Scones, cake or dessert | ☑ | ☑ |  |  |  |  |  |
|  | Loafs, muffins | ☑ | ☑ |  |  |  |  |  |
|  | Slices, friands | ☑ | ☑ |  |  |  |  |  |
|  | Biscuits, muesli bars, pikelets | ☑ | ☑ |  |  |  |  |  |
|  | Pies and quiches |  | ☑ |  |  |  |  |  |
|  | Small pastries | ☑ | ☑ |  |  |  |  |  |
|  | Sausage rolls |  | ☑ |  |  |  |  |  |
| Cold drinks | Water (plain and carbonated) |  |  |  |  |  |  |  |
|  | Water (flavoured, sweetened) | ☑ | ☑ |  |  |  |  |  |
|  | Still or carbonated flavoured drinks and milk drinks | ☑ | ☑ |  |  |  |  |  |
|  | Fruit and/or vegetable juices | ☑ | ☑ |  | ☑ |  |  |  |
|  | Ice blocks | ☑ | ☑ |  |  |  |  |  |
|  | Coconut water | ☑ | ☑ |  |  |  |  |  |
|  | Energy drinks |  |  |  |  |  |  |  |
| Milk-based smoothies prepared on site |  | ☑ | ☑ |  |  |  |  |  |

^1^HSR, Health Star Rating; a voluntary front-of-pack labelling system used in Australia and New Zealand, packaged foods and drinks are rated from 0.5 to 5 stars based on their energy, protein, total sugar, saturated fat, and sodium content, and % of fruits, vegetables, nuts, legumes and seeds in the product.
